# sgRNA Knock-in Mouse Provides an Alternative Approach for *In Vivo* Genetic Modification

**DOI:** 10.3389/fcell.2021.769673

**Published:** 2022-01-18

**Authors:** Lichen Zhang, Wushan Li, Zhuangzhuang Liu, Yang Liu, Zhilong Liu, Yanrong Gu, Le He, Binhui Zhou, Tianhan Li, Tianzhu Chao, Yinming Liang, Liaoxun Lu

**Affiliations:** ^1^ Laboratory of Genetic Regulators in the Immune System, Henan Collaborative Innovation Center of Molecular Diagnosis and Laboratory Medicine, School of Laboratory Medicine, Xinxiang Medical University, Xinxiang, China; ^2^ Henan Key Laboratory of Immunology and Targeted Therapy, Xinxiang Medical University, Xinxiang, China; ^3^ Institute of Psychiatry and Neuroscience, Xinxiang Medical University, Xinxiang, China

**Keywords:** sgRNA knock-in, mouse model, genetic modification, Gfi1, neutrophil

## Abstract

Functional genomics in a mammalian model such as mice is fundamental for understanding human biology. The CRISPR/Cas9 system dramatically changed the tempo of obtaining genetic mouse models due to high efficiency. However, experimental evidence for the establishment of sgRNA knock-in animals and analyses of their value in functional genomics are still not sufficient, particularly in mammalian models. In this study, we demonstrate that the establishment of sgRNA knock-in mice is feasible, and more importantly, crosses between sgRNA knock-in mice and the Cas9 constitutively expressing mice result in complete deletion of the target gene. Such sgRNA knock-in provides an alternative approach for *in vivo* genetic modification and can be useful in multiple circumstances, such as maintenance of genetically modified animals, which are difficult to breed as homozygotes, and cross of such mice to diverse genomic backgrounds to obtain genetically modified animals.

## Introduction

Mouse models are among the most frequently used model animals in understanding human biology. The CRISPR/Cas9 system revolutionizes accessibility to genetically modified mouse models (Komor et al., 2017). Genetic deletion of essential genes, which are highly represented in mammalian genomes, can lead to the lethality of animals or physiological abnormality such as immune deficiency. Such animals are valuable tools but are challenging to maintain as homozygous mutants. The CRISPR/Cas9 system is very intensively used to generate knockout and knock-in mouse models (Platt et al., 2014; Chu et al., 2016), and Cas9-expressing mice are broadly used to perform somatic mutations *in vivo* and *in vitro* by delivery of single-guide RNA (sgRNA) (Jinek et al., 2012; Cortez et al., 2020). However, mouse models genetically modified to express sgRNA are still limited. It is tempting to assess the possibility of establishing sgRNA knock-in mice to target gene of interest and keep such knock-in mice as a stable parental line. As Cas9 nuclease may function to cleave double-strand DNA when coupled to sgRNA, it is reasonable to cross the well-established Cas9-expressing line such as Rosa26-Cas9 knock-in mice (Platt et al., 2014) to the sgRNA-expressing knock-in mice. Both Cas9- and sgRNA-expressing mice can be maintained with the intact genome, without deficiency in the gene of interest, therefore allowing for rapid expansion of the colony.

To validate such a concept, we first established sgRNA-expressing mice by knock-in insertion at the *Rosa26* locus and detected early-stage expression of sgRNA in the embryos to target Gfi1, which is an essential gene for neutrophil development. It is important to note that neutrophil deficient mouse models are rare and difficult to maintain even though such a crucial cell subset in the immune system is more and more appreciated for its importance in cancer biology and infectious diseases. As expected, both sgRNA- and Cas9-expressing mice are normal for neutrophil development. Very strikingly, when such two lines were crossed to produce progeny that carries two transgenic components, Cas9 and sgRNAs, we observed a complete loss of neutrophils in both peripheral blood and bone marrow. It is important to note that in the two-cell embryo stage, sgRNAs were functional and cut the Gfi1 locus efficiently. We analyzed the *Gfi1* targeted sequence by next-generation sequencing or NGS and found that, in the bone marrow and blood cells, the progeny carrying two transgenic components, Cas9 and sgRNAs, had over 99.00% of the total reads modified by CRISPR/Cas9. Therefore, our experiments provided an alternative approach to obtain mouse models with severe physiological deficiency by crossing two healthy parental lines: one expressing Cas9 nuclease and another expressing sgRNAs. Such an approach could also be useful to obtain mouse models with compound mutations that are challenging to maintain and expand.

## Material and Methods

### Mice

C57BL/6 mice were purchased from Vital River Laboratory Animal Technology Co., Ltd. (Beijing, China), and NOD mice were obtained from GemPharmatech Co., Ltd. (Jiangsu, China). Rosa26-Cas9 knock-in mice on B6J (designated as Rosa26-Cas9 hereafter) constitutively expressing Cas9 and EGFP in a widespread fashion under the control of a CAG promoter were obtained from the Jackson Laboratory (stock no. 026179).

### Preparation of ssDNA by *In Vitro* Transcription and Reverse Transcription

The targeting vector containing homology arms (5’HA and 3’HA) for homologous recombination and sgGfi1 expression elements (U6-Guide 1-sgRNA scaffold-pT-U6-Guide 2-sgRNA scaffold-pT) was commercially synthesized (GenScript, China). Detailed information of synthesized sequences is provided in the Supplementary Material. A T7 promoter sequence (5’- TAA​TAC​GAC​TCA​CTA​TAG​GG -3’) was placed just upstream of the 5’HA, and a BamHI site was inserted immediately downstream of the 3’HA. The targeting vector was first linearized by digestion with BamHI (New England Biolabs) and then used as a template for *in vitro* transcription using HiScribe T7 Quick High Yield RNA Synthesis Kit (New England Biolabs). Purification of the synthesized RNAs was performed using MEGAclear kit after DNase treatment (Thermo Fisher Scientific). The cDNAs were reverse-transcribed from the synthesized RNAs using SuperScript IV Reverse Transcriptase (Thermo Fisher Scientific, 18091050) with the specific primer RT-ssDNA (Supplementary Table S1) and then purified using QIAquick PCR Purification Kit (Qiagen). The concentrations of cDNAs were measured using NanoDrop™ One Microvolume UV-Vis Spectrophotometer (Thermo Fisher Scientific). The purified cDNAs were ready as ssDNA for zygote microinjection.

### Generation and Genotyping of *Rosa26* ^*U6-sgRNA-Gfi1*^ Knock-in Mice

**FIGURE 1:**
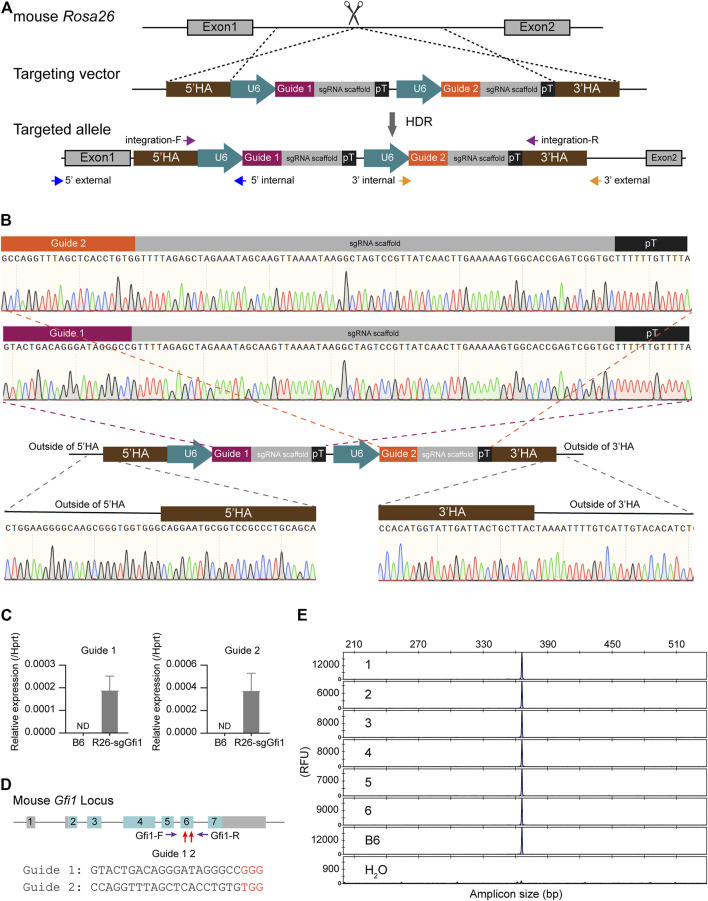
Generation of *Rosa26* ^*U6-sgRNA-Gfi1*^ knock-in mice. **(A)** Schematic diagram of knock-in of 5’HA-(U6-Guide-sgRNA scaffold-pT) × 2-3’HA vector to mouse *Rosa26* locus. U6: RNA polymerase III promoter; pT: polyT signal. **(B)** Sequencing analysis of junction-PCR products confirmed the precise insertion of the two U6-sgRNA units arrayed in tandem into the *Rosa26* locus of F0 founders. Chromatographs showed the correct sequences of the two U6-sgRNA cassettes. **(C)** Assessing the Guide 1 and Guide 2 expression in the *Rosa26* ^*U6-sgRNA-Gfi1*^ (R26-sgGfi1) embryos using real-time qPCR. B6: two-cell embryos derived from IVF using sperms and eggs of both wild-type B6 mice. ND: not detected. **(D)** Diagram of the mouse *Gfi1* locus. The Guide 1 and Guide 2 target sequences were located within exon 6 of the *Gfi1* gene. A pair of primers (Gfi1-F and Gfi1-R) spanned the entire exon 6 and gave an amplicon of 368 bp. **(E)** Representative data of capillary array electrophoresis (CAE) of R26-sgGfi1 embryos to detect genetic modification mediated by sgGfi1 *via* CRISPR/Cas9. RFU: relative fluorescent unit.

To target the *Rosa26* locus, two sgRNAs, *Rosa26*-sg1 and *Rosa26*-sg2 (Supplementary Table S2), were designed using the online tool CRISPOR (Concordet and Haeussler, 2018). Cas9 mRNA and sgRNAs were synthesized by *in vitro* transcription (IVT) as described in our previous studies (Wang et al., 2018; Huang et al., 2019). Cas9 mRNA, sgRNAs, and template DNA were mixed (at concentrations of 50 ng/μL for Cas9 mRNA, 50 ng/μL for sgRNA, and 10 ng/μL for ssDNA) and microinjected into the pronuclear of fertilized embryos (Chao et al., 2021). Survived embryos were transferred into the oviducts of pseudo-pregnant ICR mice (Yang et al., 2014). Genomic DNA from F0 newborn tails was extracted and analyzed for PCR genotyping and sequencing. The position of PCR primers used to verify the precise knock-in at the Rosa26 locus and confirm the correctness of the complete cassette is shown in Figure 1A, and their sequences are listed in Supplementary Table S1.

### Detection of Genetic Modification by Fluorescence PCR and Capillary Array Electrophoresis at Single-Base Resolution

For assessing the genome modification of CRISPR/Cas9, the *Rosa26* ^*U6-sgRNA-Gfi1*^ knock-in mice were crossed with Rosa26-Cas9 knock-in mice to generate Cas9:sgGfi1 hybrids. Genomic DNA was isolated from two-cell embryo, blastocyte, tail biopsies, liver, brain, peripheral blood, and bone marrow and used as a template for fluorescent PCR (fPCR) with 5′-fluorescein-amidite- (FAM-) labeled primer as previously described (Luo et al., 2018). The sequence of the fPCR-amplified region and the locations of the two guides targeting *Gfi1* are shown in Supplementary Figure S1. The PCR products were subjected to single-base resolution capillary array electrophoresis (CAE) on an ABI 3730 DNA analyzer, and data were analyzed by GeneMapper software v3.1 (Velasco et al., 2007; Lonowski et al., 2017).

### Next-Generation Sequencing of PCR Products in Gfi1 Targeted Mice

Next-generation sequencing (NGS) was performed for the Gfi1 targeted mice by amplifying the 368-bp DNA sequence, which harbors the two sgRNA targeting sites. The analyses were performed following the methods described in the literature (Clement et al., 2019). Briefly, purification of PCR products was performed using Zymoclean Gel Recovery Kit (D4008, Zymo Research), and DNA samples were quantitated by Qubit 2.0 Fluorometer (Thermo Fisher Scientific) before NGS library preparation using NEB DNA Library Prep Kit for Illumina (E7645). The raw data were obtained by Illumina Hiseq2500 system (PE250), and the data were processed and analyzed using CRISPResso2. Bone marrow cells and peripheral blood cells without red blood cells were used to prepare genomic DNA before PCR amplification to produce 368-bp amplicons for NGS. For each type of samples, three individual mice were used to analyze genetic modifications inside the PCR products. For each sample, around 100,000 effective reads were obtained from NGS of the PCR products. The localization of insertions, deletions, and substitutions was analyzed in a combined manner and separately. Frequencies of the indels and the reads without indels were also analyzed.

### Extraction of Small RNAs and Real-Time Quantitative PCR Analysis

Total RNA containing small RNAs were extracted from approximately 700 two-cell stage embryos using RNeasy Plus Micro Kit (Qiagen, 74034), which is designed for small amounts of cell samples and practicable for purification of small RNAs (<200 nucleotides). The first-strand cDNA was synthesized using specific primer Guide-R (Supplementary Table S1) and SuperScript IV Kit (Thermo Fisher Scientific) with random primers and gene-specific primers. 3 qPCR reactions were performed using SYBR Select Master Mix Kits (Thermo Fisher Scientific, 4472919) in 7500 Fast Real-Time PCR Systems. Mouse *Hprt* was used as a housekeeping gene. Relative gene expression was calculated by the 2^(−ΔCT)^ method. Gene-specific primers for real-time PCR are listed in Supplementary Table S1.

### Flow Cytometry Analysis

For immunophenotyping, peripheral blood cells and bone marrow cells from WT and KI mice were stained with monoclonal antibody mixes, and then cells were acquired on the Invitrogen Attune NxT Flow Cytometer (Thermo Fisher Scientific). The FACS data were analyzed using FlowJo software version 10.0. Antibodies used in this study are listed in Supplementary Table S3.

### Statistical Analysis

All data were analyzed with GraphPad Prism software (version 8.0) and presented as mean ± SEM. Statistical significance was calculated by unpaired, two-tailed Student’s *t*-test: *p < 0.05, **p < 0.01, ***p < 0.001, ns: not significant.

## Results

### Knock-in of U6-sgRNA-Gfi1 at *Rosa26* Locus in C57BL/6 Mice

To test the feasibility of sgRNA knock-in at the *Rosa26* locus in mice, we obtained a targeting vector that served as a homology-directed repair (HDR) template, including two independent sgRNAs targeting *Gfi1*, an essential gene for neutrophil development (Ordoñez-Rueda et al., 2012). As shown in Figure 1A, the donor DNA includes 5’ and 3’ homology arms and two expression cassettes of U6 promoter-sgRNA arrayed in tandem. Fertilized eggs from C57BL/6 mice were prepared *in vitro*, then subjected to microinjection with the ssDNA donor and CRISPR/Cas9 reagents, and transplanted to ICR foster mice as described in our studies (Chao et al., 2021). PCR-based genotyping of F0 mice was conducted to screen for candidate individuals, and sequencing of the entire knock-in insertion fragment, 5’- and 3’-junction regions was performed to validate the F0 founders harboring the correct targeted allele (Figure 1B). The resultant knock-in F0 mice were crossed to C57BL/6 (B6) wild-type animals for colony expansion and to keep wild-type *Gfi1* background, which were also sequenced to confirm that the correct knock-in allele was transmitted to the F1 offspring.

To determine whether the two U6-sgRNAs were expressed or not in the early developmental stage, approximately 700 two-cell embryos derived from *in vitro* fertilization (IVF) using sperms of *Rosa26* ^*U6-sgRNA-Gfi1*^ homozygous mice and WT B6 oocytes were subjected to RNA preparation and real-time qPCR for evaluating the expression of sgRNA in comparison to embryos derived from both WT B6 sperms and oocytes (Figure 1C). Both Guide 1 RNA and Guide 2 RNA showed evident expression, indicating early expression during the development of mice *via* knock-in of the tandemly arrayed U6 promoter-sgRNA units into the *Rosa26* locus. We further validated that, in the absence of Cas9 nuclease, the knock-in of *Rosa26* ^*U6-sgRNA-Gfi1*^ was not modifying *Gfi1* locus, and FAM fluorophore-labeled DNA primers were used to amplify the sgRNA targeting loci as indicated in Figure 1D. The PCR products were analyzed by single-base resolution CAE analysis to detect small insertions/deletions. Our results showed that, in the absence of Cas9 nuclease, among the 12 two-cell stage embryos derived from IVF using sperms of *Rosa26* ^*U6-sgRNA-Gfi1*^ mice and eggs of WT B6 mice, there was no detectable DNA cleavage as detected by CAE at 1-bp resolution (Figure 1E). Our experiments showed that, in the stable colony of *Rosa26* ^*U6-sgRNA-Gfi1*^ homozygous mice obtained from F0 knock-in founder and sequential backcrossing and intercrossing, we successfully established a knock-in mouse line that expresses sgRNA in early developmental stages.

### Normal Neutrophil Development in Both C57BL/6 *Rosa26* ^*U6-sgRNA-Gfi1*^ Mice and Rosa26-Cas9 Mice

**FIGURE 2:**
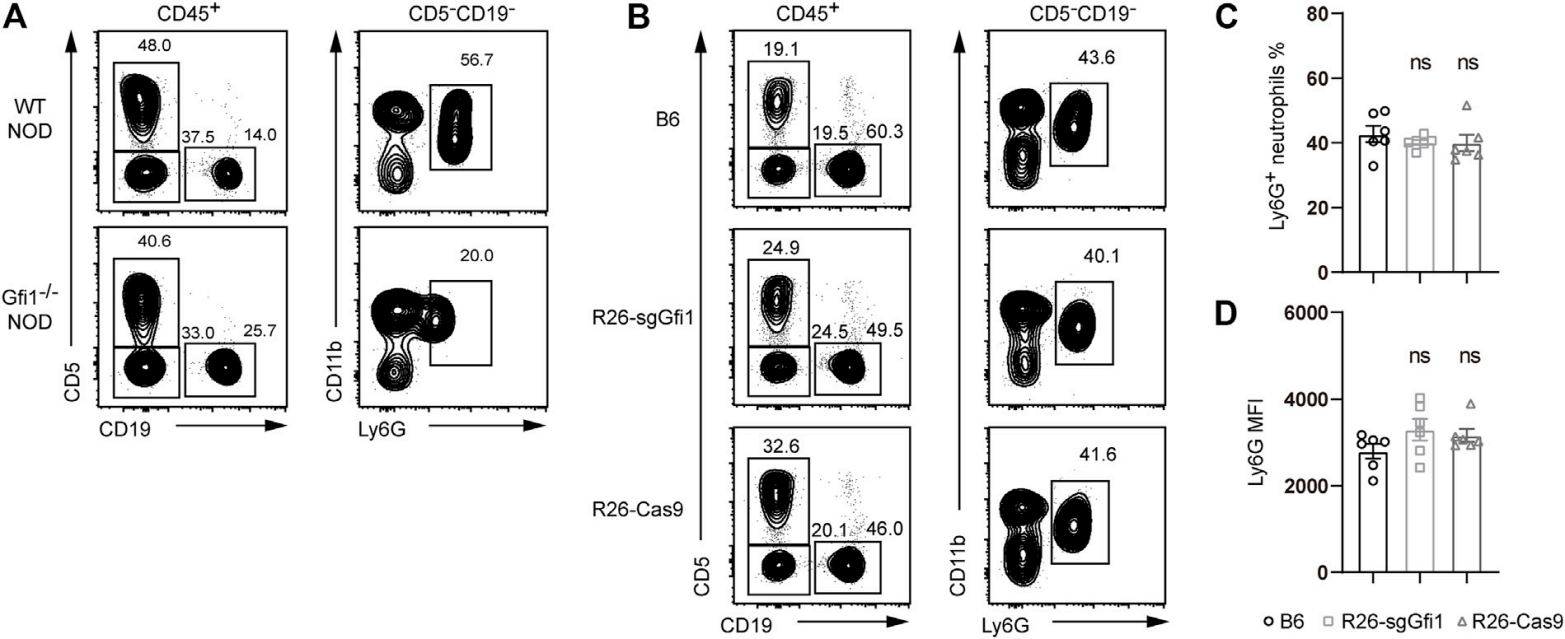
Ly6G expression in Gfi1^−/−^ NOD mice, *Rosa26* ^*U6-sgRNA-Gfi1*^, and R26-Cas9 B6 mice. **(A)** Flow cytometric analyses of peripheral blood from Gfi1^−/−^ NOD mice compared to wild-type NOD mice. White blood cells were defined by gating on CD45^+^ populations, B cells on CD19^+^, and T cells on CD5^+^; neutrophils were gated on Cd11b^+^ Ly6G^+^ cells corresponding to CD5^−^CD19^−^ nonlymphoid populations. **(B)** Flow cytometry analysis of CD11b^+^Ly6G^+^ neutrophils in peripheral blood of WT B6, *Rosa26* ^*U6-sgRNA-Gfi1*^ mice (R26-sgGfi1), and Rosa26-Cas9 mice (R26-Cas9). **(C–D)** Percentages **(C)** and mean fluorescence intensity (MFI) **(D)** of CD11b^+^Ly6G^+^ neutrophils in peripheral blood of WT B6, R26-sgGfi1, and R26-Cas9 mice, mean ± SEM, *n* = 6.

Gfi1 is crucial for neutrophil development, and mutations of Gfi1 could result in an obvious phenotype of neutrophil reduction or absence in peripheral blood on the C57BL/6 background. We also performed CRISPR/Cas9-mediated gene deletion on the NOD background, which possesses the largest amounts of polymorphisms among all the inbred strains and displays dramatically different phenotypes in immunity (McClive et al., 1994; Wang et al., 2018). As shown in Figure 2A, we performed CRISPR/Cas9-mediated knockout of *Gfi1* in NOD mice, and we observed loss of mature circulating neutrophils, which was consistent with the phenotype reported in C57BL/6 mice (Ordoñez-Rueda et al., 2012). Neutrophils are broadly involved in host defense, and they are immune cells of great interest for cancer biology and infectious diseases (Furumaya et al., 2020; Lehman and Segal, 2020). However, neutrophil deficient mouse models are rare, and they are difficult to maintain. In this study, we aimed to set up crosses between C57BL/6 *Rosa26* ^*U6-sgRNA-Gfi1*^ mice and Rosa26-Cas9 mice, which constitutively express Cas9 protein (Platt et al., 2014). In comparison to C57BL/6 wild-type mice, both C57BL/6 *Rosa26* ^*U6-sgRNA-Gfi1*^ mice and Rosa26-Cas9 mice were analyzed to quantitate neutrophils by gating the CD11b and Ly6G positive cells in peripheral blood, and we found that both C57BL/6 *Rosa26* ^*U6-sgRNA-Gfi1*^ mice and Rosa26-Cas9 mice had normal neutrophils (Figures 2B,C). As Ly6G is a specific marker for neutrophil and its expression reflects neutrophil maturation (Hasenberg et al., 2015; Chao et al., 2021), we analyzed the surface expression of Ly6G using mean fluorescence intensity by flow cytometry and found that the expression levels of Ly6G were comparable between different groups of mice (Figure 2D). Taking together our immunophenotyping data from flow cytometric analyses, we confirmed that both C57BL/6 *Rosa26* ^*U6-sgRNA-Gfi1*^ mice and Rosa26-Cas9 mice had normal development of neutrophils.

### Efficient Gfi1 Deletion in Progeny Derived From Crossing Between C57BL/6*Rosa26* ^*U6-sgRNA-Gfi1*^ and Rosa26-Cas9 Mice

**FIGURE 3:**
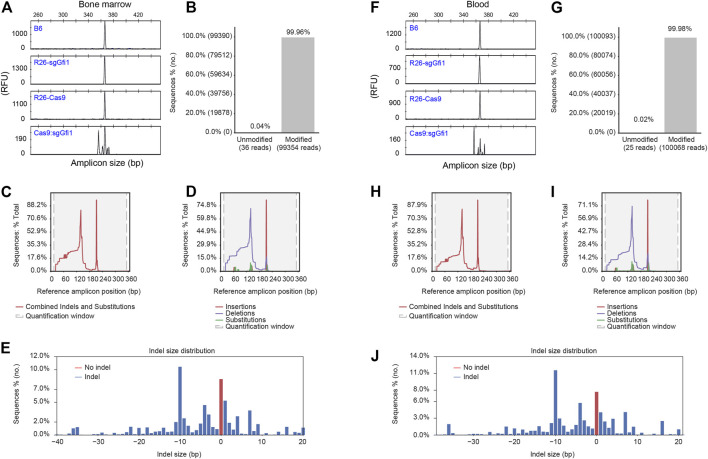
Detection of CRISPR/Cas9-mediated genetic modification of *Gfi1* gene. **(A)** Bone marrow cells were isolated and subjected to red blood cell lysis before extraction of genomic DNA and PCR amplification using 5’FAM labeled oligonucleotide. The PCR products from Gfi1 targeted Cas9:sgGfi1 mice and control mice were analyzed by fPCR-CAE. **(B)** Bone marrow cells were used for the analysis of DNA modification in Gfi1 targeted Cas9:sgGfi1 mice. The same PCR primers as used in **(A)** without 5’FAM labeling were used to amplify Gfi1 targeted Cas9:sgGfi1 mice, and the PCR products were analyzed by NGS to detect both unmodified and modified sequences by alignment of the reads to a wild-type reference. **(C)** Bone marrow cells were used for combined analysis of the localization of the DNA modifications, which included insertions, deletions, and substitutions. **(D)** Bone marrow cells were used for the analysis of the localization of the DNA modifications in a separate manner, which included insertions, deletions, and substitutions, respectively. **(E)** Bone marrow cells were used for frequency analysis of indels with difference sizes occurring at the targeted Gfi1 locus, and the red bar represents reads without size change. **(F–J)** Cells from peripheral blood were used for genomic DNA preparation, and the analyses of Gfi1 locus in the targeted mice were performed in the same way as the bone marrow samples. WT B6, *Rosa26* ^*U6-sgRNA-Gfi1*^ (Rosa26-sgGfi1), and Rosa26-Cas9 mice were used as negative controls for **(A,F)**. RFU: relative fluorescent unit.

Since both C57BL/6 *Rosa26* ^*U6-sgRNA-Gfi1*^ mice and Rosa26-Cas9 mice have normal neutrophils, we further crossed these two lines to have mice expressing Cas9 nuclease and sgRNAs *in vivo*. As described above, sgRNA expression can be detected at two-cell stage embryos. Therefore, it is possible to obtain progeny from crosses between C57BL/6 *Rosa26* ^*U6-sgRNA-Gfi1*^ and Rosa26-Cas9 mice, which have Gfi1 deleted in the early stage of development. If this is true, we could obtain F1 hybrids from crosses between C57BL/6 *Rosa26* ^*U6-sgRNA-Gfi1*^ and Rosa26-Cas9 mice that can be immediately phenotyped as Gfi1 deficient mutants. It is important to note that such F1 hybrids are derived from healthy parental mice without neutrophil deficiency, as described above. To detect DNA cleavage at *Gfi1* locus during early stages, a single embryo was analyzed 5 d after IVF. In such embryos derived from zygotes injected with Cas9 mRNA using sperms from *Rosa26* ^*U6-sgRNA-Gfi1*^ mice and eggs from WT mice, we found that 16 out of 18 displayed evident DNA cutting by CRISPR/Cas9 although the wild-type allele was still present (Supplementary Figure S2A). Such results showed that sgRNA expression in the early stage of development was functional and sufficient to cut double-strand DNA when coupled to Cas9 nuclease. We further analyzed DNA cleavage in tissues using F1 progeny from crosses between C57BL/6 *Rosa26* ^*U6-sgRNA-Gfi1*^ and Rosa26-Cas9 mice (hereinafter referred to as Cas9:sgGfi1). We applied CAE to assess the efficiency of CRISPR/Cas9 in the deletion of Gfi1 *in vivo*. Interestingly, we found that, in all the tissues assayed, wild-type sized PCR amplicons of Gfi1 were rarely detectable (Figures 3A,F; Supplementary Figures S2B–D); except for that in bone marrow cells, the wild-type sized PCR products were noticeable (Figure 3A). It is important to note that such size changes detected by CAE at 1-bp resolution were not observed in WT B6, *Rosa26* ^*U6-sgRNA-Gfi1*^, or Rosa26-Cas9 mice.

In further experiments, we aimed for a more in-depth analysis of such PCR products from Gfi1 targeted mice by NGS. Strikingly, in all the 6 samples, including 3 bone marrow samples and 3 blood samples, we observed 99.90–99.98% of the amplified sequences were modified in the Cas9:sgGfi1 mice (Figures 3B,G; Supplementary Figure S3). For combined analyses of the localization of the genetic modifications inside the amplicons, which include insertions, deletions, and substitutions, we found two discrete peaks in the histogram as shown in Figures 3C,H. In separated analyses, our data showed that deletions are distributed more broadly than insertions and substitutions (Figures 3D,I). Quite interestingly, in the bone marrow sample, the frequency of amplicons without indels or change of the size was close to 10% among the total reads (Figure 3E), even though the unmodified reads of this bone marrow sample were as low as 0.04% (Figure 3B). Such data indicate that the substitutions altogether accounted for close to 10% of the genetic modifications. We applied parallel analyses using the blood samples and obtained similar results (Figures 3G–J). Taken together, the NGS data showed that the modification by CRISPR/Cas9 in Cas9:sgGfi1 mice was thorough, even though such modification involved a noticeable amount of substitution mutations. Discrete distribution of the histogram showing localization of the genetic modifications, including insertions, deletions, and substitution, indicated that two sgRNA were functional *in vivo*. Therefore, by crossing C57BL/6 *Rosa26* ^*U6-sgRNA-Gfi1*^ and Rosa26-Cas9 mice, we obtained the F1 Cas9:sgGfi1 mice, which had early onset of deletion of Gfi1, and in multiple tissues, Gfi1 deletion was evident. Except for the bone marrow, in all the other tissues analyzed, the wild-type sized PCR products were hardly detectable.

### Loss of Neutrophils in Cas9:sgGfi1 Mice Expressing Both sgRNAs and Cas9 Nuclease *In Vivo*

**FIGURE 4:**
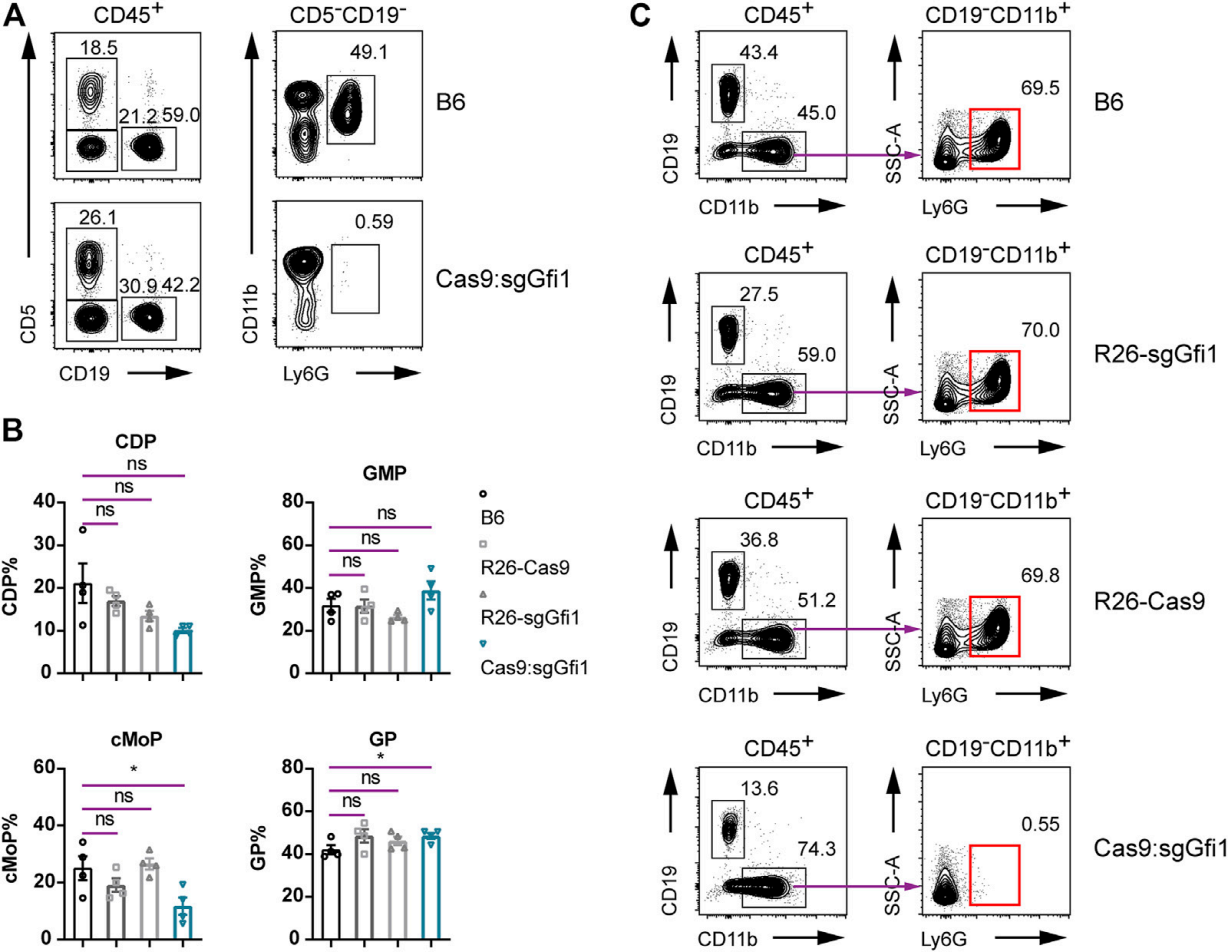
Immunophenotyping of Cas9:sgGfi1 mice. **(A)** Determination of circulating neutrophils in peripheral blood from Cas9:sgGfi1 mice analyzed by flow cytometry. The gating strategy is the same as that used in Figure 2A. **(B)** Percentages of BM-derived myeloid progenitors CDP, GMP, cMoP, and GP of WT B6, *Rosa26* ^*U6-sgRNA-Gfi1*^ mice (R26-sgGfi1), Rosa26-Cas9 mice (R26-Cas9), and Cas9:sgGfi1 mice, mean ± SEM, *n* = 4. CDP: common DC progenitor; GMP: granulocyte-monocyte progenitor; cMoP: common monocyte progenitor; GP: granulocyte committed progenitor. **(C)** Representative flow cytometric analysis of Ly6G^+^ neutrophils in the bone marrow of WT B6, Rosa26-sgGfi1, Rosa26-Cas9, and Cas9:sgGfi1 mice.

In the progeny derived from crosses between C57BL/6 *Rosa26* ^*U6-sgRNA-Gfi1*^ and Rosa26-Cas9 mice, it was evident that Gfi1 deletion did occur *in vivo* and over 99.9% of the bone marrow cells had genetic modifications, regardless of the difficulties in differentiating DNA cutting efficiency during neutrophil differentiation from early progenitors. To analyze the phenotype of neutrophils in Cas9:sgGfi1 mice that express both sgRNA targeting Gfi1 and the Cas9 nuclease, we performed flow cytometric analyses using peripheral blood. Very strikingly, we observed a complete loss of neutrophils in the peripheral blood of Cas9:sgGfi1 mice (Figure 4A). Since neutrophil development is dependent on granulopoiesis in bone marrow, which requires differentiation of progenitor cells residing in the bone marrow, we further analyzed the neutrophils, as well as their progenitors in the bone marrow. Our experiments found that the frequencies of granulocyte progenitor were not obviously affected, even though we noticed a significant decrease in cMoP, a subset of monocyte progenitors (Figure 4B). We, therefore, sought to analyze the neutrophils in the bone marrow. In the control groups, all the mice had normal development of neutrophils in the bone marrow. However, in the Cas9:sgGfi1 mice, which express both sgRNA targeting Gfi1 and the Cas9 nuclease, we observed a complete loss of Ly6G^+^ neutrophils in bone marrow (Figure 4C). Such intriguing results suggest that Gfi1 deficiency may occur before maturation of Ly6G^+^ neutrophils regardless of the fact their progenitor cells were not affected in frequencies, but such progenitors could be compromised in capacity for cell differentiation and maturation. Such an experiment showed that neutrophil sufficient parental mice, namely, the C57BL/6 *Rosa26* ^*U6-sgRNA-Gfi1*^ and Rosa26-Cas9 mice, can be used to produce neutrophil deficient mice due to early onset of DNA cutting and thorough gene deletion mediated by CRISPR/Cas9 *in vivo* before neutrophil maturation. Therefore, our experiments provided an alternative approach to maintain a colony of fragile but valuable mouse models such as neutrophil deficient mice. Additionally, this study suggests that at least two sgRNAs can be simultaneously functional *in vivo* in knock-in mice.

## Discussion

CRISPR/Cas9 genome editing tool has been successfully applied to establish genetic mouse models. To do germ-line mutation, microinjection of ribonucleoprotein (RNP), consisting of the Cas9 protein in complex with targeting gRNAs into fertilized eggs, yields mutant mice in high efficiency. To obtain tissue-specific mutant and somatic mutations, CRISPR/Cas9 has been successfully and widely used to establish mouse models harboring Cre/loxP system. For neurobiology, it is regular to deliver sgRNA *via* viral vectors into the Cas9 mice, which express Cas9 nuclease constitutively or in an inducible manner as Cas9 expression can be controlled by the presence of Cre recombinase (Madisen et al., 2010; Daigle et al., 2018). However, the sgRNA-expressing mice constructed and maintained as stable lines are still limited, even though various scenarios requiring genetically modified mice may need such a tool. At present, to obtain genetically deficient mice as homozygous is only feasibly for non-essential genes in terms of survival and fertility. It is regular to cross heterozygous animals for genotyping and selection of homozygous mutants, which is limited by the efficiency due to the Mendelian law of inheritance. For such a practice to cross sgRNA-expressing mice to Cas9-expressing mice, which could result in a loss-of-function mutation in a certain gene, several technical issues have to be solved, which may first involve the establishment of sgRNA knock-in mice and validation of the efficiency genetic modification *in vivo* by crossing them to Cas9 nuclease expressing mice. In our experiments, we aimed to establish an alternative neutrophil deficient model. We first obtained a stable colony of *Rosa26* ^*U6-sgRNA-Gfi1*^ homozygous mice obtained from F0 knock-in founder and sequential backcrossing and intercrossing to keep Gfi1 wild-type background. Such a knock-in line expresses sgRNA in early developmental stages, which could allow for germ-line deletion of genes when it is crossed to Rosa26-Cas9-expressing mice. The progeny from such crosses led to the early-stage deletion of Gfi1 in embryos, and genetic analyses of multiple tissues showed that Gfi1 deletion was global and efficient. Further, this technical study showed that progeny from crosses between C57BL/6 *Rosa26* ^*U6-sgRNA-Gfi1*^ and Rosa26-Cas9 mice had a complete absence of neutrophils. We observed a complete loss of Ly6G^+^ neutrophils in this Cas9:sgGfi1 model, which is different from the *Gfi1* ^*C318Y*^ point mutation model obtained from N-ethyl-N-nitrosourea or ENU mutagenesis (Ordoñez-Rueda et al., 2012) with partial loss of neutrophils. The absence of neutrophil in this Cas9:sgGfi1 is even more complete than the conventional Gfi1 knockout model on NOD background, which suggests the high efficiency of sgRNA targeting in the presence of Cas9 nuclease *in vivo*. Therefore, our experiments proved the concept to establish genetically deficient mouse models from healthy sgRNA-expressing and Cas9-expressing parental lines, which provided an alternative approach to maintain colony under challenging situations such as loss of immune protection or infertility causing haploinsufficiency (Tan et al., 2020). In addition, our experiments suggested the possibility of establishing a mouse model with compound mutations as more than one sgRNA can be introduced during the knock-in process. Based on the extremely high efficiency of *in vivo* genetic modification revealed by NGS and robust mutant phenotype in such Cas9:sgGfi1 mice, one sgRNA could be sufficient when one gene is targeted. The discrete localizations of genetic modifications as shown in the histogram of combined analyses of the deletions, insertions, and substitutions indicated comparable efficiencies of two independent sgRNAs. Therefore, knock-in expression of two or more sgRNAs can achieve compound mutations when double knockout or more sophisticated genetic modification *in vivo* is required. It is also important to note that a wide-type sized PCR product of the targeted locus included a noticeable amount of substitution mutations that can still result in deleterious effects; NGS analysis of the targeted sites in such mice is necessary.

Our current study is limited to the constitutive deletion of a target gene. In this case, Gfi1, an essential gene for neutrophil development, and conditional manipulation using inducible Cas9 could be further investigated. Our current study generated a successful model with a neutrophil deficiency, which is less challenging to maintain and expand since both C57BL/6 *Rosa26* ^*U6-sgRNA-Gfi1*^ and Rosa26-Cas9 mice are healthy and fertile. Therefore, our experiments have proven that sgRNA-expressing mice can be generated by CRISPR/Cas9 and maintained as a stable line, which can be crossed to Rosa26-Cas9 mice. Crossing healthy *Rosa26* ^*U6-sgRNA-Gfi1*^ and Rosa26-Cas9 mice resulted in complete penetrance of the F1 hybrids, which are completely derived from neutrophils.

## Data Availability

The datasets presented in this study can be found in online repositories. The names of the repository/repositories and accession number(s) can be found below: NCBI SRA BioProject, accession no: PRJNA791773.

## References

[B1] Chao T. Lu L. Zhang L. Huang R. Liu Z. Zhou B. (2021). An Inducible Model for Specific Neutrophil Depletion by Diphtheria Toxin in Mice. Sci. China Life Sci. 64, 1227–1235. 10.1007/s11427-020-1839-3 33420927

[B2] Chu V. T. Graf R. Wirtz T. Weber T. Favret J. Li X. (2016). Efficient CRISPR-Mediated Mutagenesis in Primary Immune Cells Using CrispRGold and a C57BL/6 Cas9 Transgenic Mouse Line. Proc. Natl. Acad. Sci. USA 113, 12514–12519. 10.1073/pnas.1613884113 27729526 PMC5098665

[B3] Clement K. Rees H. Canver M. C. Gehrke J. M. Farouni R. Hsu J. Y. (2019). CRISPResso2 Provides Accurate and Rapid Genome Editing Sequence Analysis. Nat. Biotechnol. 37 (3), 224–226. 10.1038/s41587-019-0032-3 30809026 PMC6533916

[B4] Concordet J.-P. Haeussler M. (2018). CRISPOR: Intuitive Guide Selection for CRISPR/Cas9 Genome Editing Experiments and Screens. Nucleic Acids Res. 46, W242–W245. 10.1093/nar/gky354 29762716 PMC6030908

[B5] Cortez J. T. Montauti E. Shifrut E. Gatchalian J. Zhang Y. Shaked O. (2020). CRISPR Screen in Regulatory T Cells Reveals Modulators of Foxp3. Nature 582, 416–420. 10.1038/s41586-020-2246-4 32499641 PMC7305989

[B6] Daigle T. L. Madisen L. Hage T. A. Valley M. T. Knoblich U. Larsen R. S. (2018). A Suite of Transgenic Driver and Reporter Mouse Lines with Enhanced Brain-cell-type Targeting and Functionality. Cell 174, 465–480. 10.1016/j.cell.2018.06.035 30007418 PMC6086366

[B7] Furumaya C. Martinez-Sanz P. Bouti P. Kuijpers T. W. Matlung H. L. (2020). Plasticity in Pro- and Anti-tumor Activity of Neutrophils: Shifting the Balance. Front. Immunol. 11. 10.3389/fimmu.2020.02100 PMC7492657 32983165

[B8] Hasenberg A. Hasenberg M. Männ L. Neumann F. Borkenstein L. Stecher M. (2015). Catchup: a Mouse Model for Imaging-Based Tracking and Modulation of Neutrophil Granulocytes. Nat. Methods 12, 445–452. 10.1038/nmeth.3322 25775045

[B9] Huang R. Guo G. Lu L. Fu R. Luo J. Liu Z. (2019). The Three Members of the Vav Family Proteins Form Complexes that Concur to Foam Cell Formation and Atherosclerosis. J. Lipid Res. 60, 2006–2019. 10.1194/jlr.M094771 31570505 PMC6889716

[B10] Jinek M. Chylinski K. Fonfara I. Hauer M. Doudna J. A. Charpentier E. (2012). A Programmable Dual-RNA-Guided DNA Endonuclease in Adaptive Bacterial Immunity. Science 337, 816–821. 10.1126/science.1225829 22745249 PMC6286148

[B11] Komor A. C. Badran A. H. Liu D. R. (2017). CRISPR-based Technologies for the Manipulation of Eukaryotic Genomes. Cell 168, 20–36. 10.1016/j.cell.2016.10.044 27866654 PMC5235943

[B12] Lehman H. K. Segal B. H. (2020). The Role of Neutrophils in Host Defense and Disease. J. Allergy Clin. Immunol. 145, 1535–1544. 10.1016/j.jaci.2020.02.038 32283205 PMC8912989

[B14] Lonowski L. A. Narimatsu Y. Riaz A. Delay C. E. Yang Z. Niola F. (2017). Genome Editing Using FACS Enrichment of Nuclease-Expressing Cells and Indel Detection by Amplicon Analysis. Nat. Protoc. 12, 581–603. 10.1038/nprot.2016.165 28207001 PMC7250141

[B15] Luo J. Lu L. Gu Y. Huang R. Gui L. Li S. (2018). Speed Genome Editing by Transient CRISPR/Cas9 Targeting and Large DNA Fragment Deletion. J. Biotechnol. 281, 11–20. 10.1016/j.jbiotec.2018.06.308 29886029

[B16] Madisen L. Zwingman T. A. Sunkin S. M. Oh S. W. Zariwala H. A. Gu H. (2010). A Robust and High-Throughput Cre Reporting and Characterization System for the Whole Mouse Brain. Nat. Neurosci. 13, 133–140. 10.1038/nn.2467 20023653 PMC2840225

[B17] Mcclive P. J. Baxter A. Morahan G. (1994). Genetic Polymorphisms of the Non-obese Diabetic (NOD) Mouse. Immunol. Cel Biol 72, 137–142. 10.1038/icb.1994.21 7911121

[B18] Ordoñez-Rueda D. Jönsson F. Mancardi D. A. Zhao W. Malzac A. Liang Y. (2012). A Hypomorphic Mutation in the Gfi1 Transcriptional Repressor Results in a Novel Form of Neutropenia. Eur. J. Immunol. 42, 2395–2408. 10.1002/eji.201242589 22684987

[B19] Platt R. J. Chen S. Zhou Y. Yim M. J. Swiech L. Kempton H. R. (2014). CRISPR-Cas9 Knockin Mice for Genome Editing and Cancer Modeling. Cell 159, 440–455. 10.1016/j.cell.2014.09.014 25263330 PMC4265475

[B20] Tan J. H. L. Wollmann H. Van Pelt A. M. M. Kaldis P. Messerschmidt D. M. (2020). Infertility-Causing Haploinsufficiency Reveals TRIM28/KAP1 Requirement in Spermatogonia. Stem Cel Rep. 14, 818–827. 10.1016/j.stemcr.2020.03.013 PMC7220855 32302554

[B21] Velasco E. Infante M. Durán M. Pérez-Cabornero L. Sanz D. J. Esteban-Cardeñosa E. (2007). Heteroduplex Analysis by Capillary Array Electrophoresis for Rapid Mutation Detection in Large Multiexon Genes. Nat. Protoc. 2, 237–246. 10.1038/nprot.2006.482 17401359

[B22] Wang X. Huang R. Zhang L. Li S. Luo J. Gu Y. (2018). A Severe Atherosclerosis Mouse Model on the Resistant NOD Background. Dis. Model. Mech. 11. 10.1242/dmm.033852 PMC6215432 30305306

[B23] Yang H. Wang H. Jaenisch R. (2014). Generating Genetically Modified Mice Using CRISPR/Cas-mediated Genome Engineering. Nat. Protoc. 9, 1956–1968. 10.1038/nprot.2014.134 25058643

