# Supplementary material for: sgRNA Knock-in Mouse Provides an Alternative Approach for In Vivo Genetic Modification
Source: Front Cell Dev Biol. 2022 Jan 18;9:769673. doi: 10.3389/fcell.2021.769673 (PMC8804526; doi:10.3389/fcell.2021.769673)
Supplement: Supplementary file 1 [file DataSheet1.docx]

Supplementary Material

# Supplementary Data | Sequence of the homologous recombination template for knock-in of *Rosa26^U6-sgRNA-Gfi1^*.

**>5’** **homology arms (HA)**

CAGGAATGCGGTCCGCCCTGCAGCAACCGGAGGGGGAGGGAGAAGGGAGCGGAAAAGTCTCCACCGGACGCGGCCATGGCTCGGGGGGGGGGGGGCAGCGGAGGAGCGCTTCCGGCCGACGTCTCGTCGCTGATTGGCTTCTTTTCCTCCCGCCGTGTGTGAAAACACAAATGGCGTGTTTTGGTTGGCGTAAGGCGCCTGTCAGTTAACGGCAGCCGGAGTGCGCAGCCGCCGGCAGCCTCGCTCTGCCCACTGGGTGGGGCGGGAGGTAGGTGGGGTGAGGCGAGCTGGACGTGCGGGCGCGGTCGGCCTCTGGCGGGGCGGGGGAGGGGAGGGAGGGTCAGCGAAAGTAGCTCGCGCGCGAGCGGCCGCCCACCCTCCCCTTCCTCTGGGGGAGTCGTTTTACCCGCCGCCGGCCGGGCCTCGTCGTCTGATTGGCTCTCGGGGCCCAGAAAACTGGCCCTTGCCATTGGCTCGTGTTCGTGCAAGTTGAGTCCATCCGCCGGCCAGCGGGGGCGGCGAGGAGGCGCTCCCAGGTTCCGGCCCTCCCCTCGGCCCCGCGCCGCAGAGTCTGGCCGCGCGCCCCTGCGCAACGTGGCAGGAAGCGCGCGCTGGGGGCGGGGACGGGCAGTAGGGCTGAGCGGCTGCGGGGCGGGTGCAAGCACGTTTCCGACTTGAGTTGCCTCAAGAGGGGCGTGCTGAGCCAGACCTCCATCGCGCACTCCGGGGAGTGGAGGGAAGGAGCGAGGGCTCAGTTGGGCTGTTTTGGAGGCAGGAAGCACTTGCTCTCCCAAAGTCGCTCTGAGTTGTTATCAGTAAGGGAGCTGCAGTGGAGTAGGCGGGGAGAAGGCCGCACCCTTCTCCGGAGGGGGGAGGGGAGTGTTGCAATACCTTTCTGGGAGTTCTCTGCTGCCTCCTGGCTTCTGAGGACCGCCCTGGGCCTGGGAGAATCCCTTCCCCCTCTTCCCTCGTGATCTGCAACTCAAGTCTTTCTAGAAGA

**>(U6-Guide-sgRNA scaffold-pT) × 2**

CTAGGAGGGCCTATTTCCCATGATTCCTTCATATTTGCATATACGATACAAGGCTGTTAGAGAGATAATTGGAATTAATTTGACTGTAAACACAAAGATATTAGTACAAAATACGTGACGTAGAAAGTAATAATTTCTTGGGTAGTTTGCAGTTTTAAAATTATGTTTTAAAATGGACTATCATATGCTTACCGTAACTTGAAAGTATTTCGATTTCTTGGCTTTATATATCTTGTGGAAAGGACGAAACACCGTACTGACAGGGATAGGGCCGTTTTAGAGCTAGAAATAGCAAGTTAAAATAAGGCTAGTCCGTTATCAACTTGAAAAAGTGGCACCGAGTCGGTGCTTTTTTGTTTTAGAGCTAGAGGTCGAGGGCCTATTTCCCATGATTCCTTCATATTTGCATATACGATACAAGGCTGTTAGAGAGATAATTGGAATTAATTTGACTGTAAACACAAAGATATTAGTACAAAATACGTGACGTAGAAAGTAATAATTTCTTGGGTAGTTTGCAGTTTTAAAATTATGTTTTAAAATGGACTATCATATGCTTACCGTAACTTGAAAGTATTTCGATTTCTTGGCTTTATATATCTTGTGGAAAGGACGAAACACCGCCAGGTTTAGCTCACCTGTGGTTTTAGAGCTAGAAATAGCAAGTTAAAATAAGGCTAGTCCGTTATCAACTTGAAAAAGTGGCACCGAGTCGGTGCTTTTTTGTTTTAGAGCTAGA

**Annotations:**

NNN: U6 promoter

NNN: Guide-1 targeting *Gfi1*

NNN: Guide-2 targeting *Gfi1*

NNN: sgRNA scaffold

NNN: polyT signal

**>3’ homology arms (HA)**

GGCGGGAGTCTTCTAGGCAGGCTTAAAGGCTAACCTGGTGTGTGGGCGTTGTCCTGCAGGGGAATTGAACAGGTGTAAAATTGGAGGGACAAGACTTCCCACAGATTTTCGGTTTTGTCGGGAAGTTTTTTAATAGGGGCAAATAAGGAAAATGGGAGGATAGGTAGTCATCTGGGGTTTTATGCAGCAAAACTACAGGTTATTATTGCTTGTGATCCGCCTCGGAGTATTTTCCATCGAGGTAGATTAAAGACATGCTCACCCGAGTTTTATACTCTCCTGCTTGAGATCCTTACTACAGTATGAAATTACAGTGTCGCGAGTTAGACTATGTAAGCAGAATTTTAATCATTTTTAAAGAGCCCAGTACTTCATATCCATTTCTCCCGCTCCTTCTGCAGCCTTATCAAAAGGTATTTTAGAACACTCATTTTAGCCCCATTTTCATTTATTATACTGGCTTATCCAACCCCTAGACAGAGCATTGGCATTTTCCCTTTCCTGATCTTAGAAGTCTGATGACTCATGAAACCAGACAGATTAGTTACATACACCACAAATCGAGGCTGTAGCTGGGGCCTCAACACTGCAGTTCTTTTATAACTCCTTAGTACACTTTTTGTTGATCCTTTGCCTTGATCCTTAATTTTCAGTGTCTATCACCTCTCCCGTCAGGTGGTGTTCCACATTTGGGCCTATTCTCAGTCCAGGGAGTTTTACAACAATAGATGTATTGAGAATCCAACCTAAAGCTTAACTTTCCACTCCCATGAATGCCTCTCTCCTTTTTCTCCATTTATAAACTGAGCTATTAACCATTAATGGTTTCCAGGTGGATGTCTCCTCCCCCAATATTACCTGATGTATCTTACATATTGCCAGGCTGATATTTTAAGACATTAAAAGGTATATTTCATTATTGAGCCACATGGTATTGATTACTGCTTACTAAAATTTTGTCATTGTACACATCTGTAAAAGGTGGTTCCTTTTGGAATGC

# Supplementary Figures and Tables

## Supplementary Figure 1


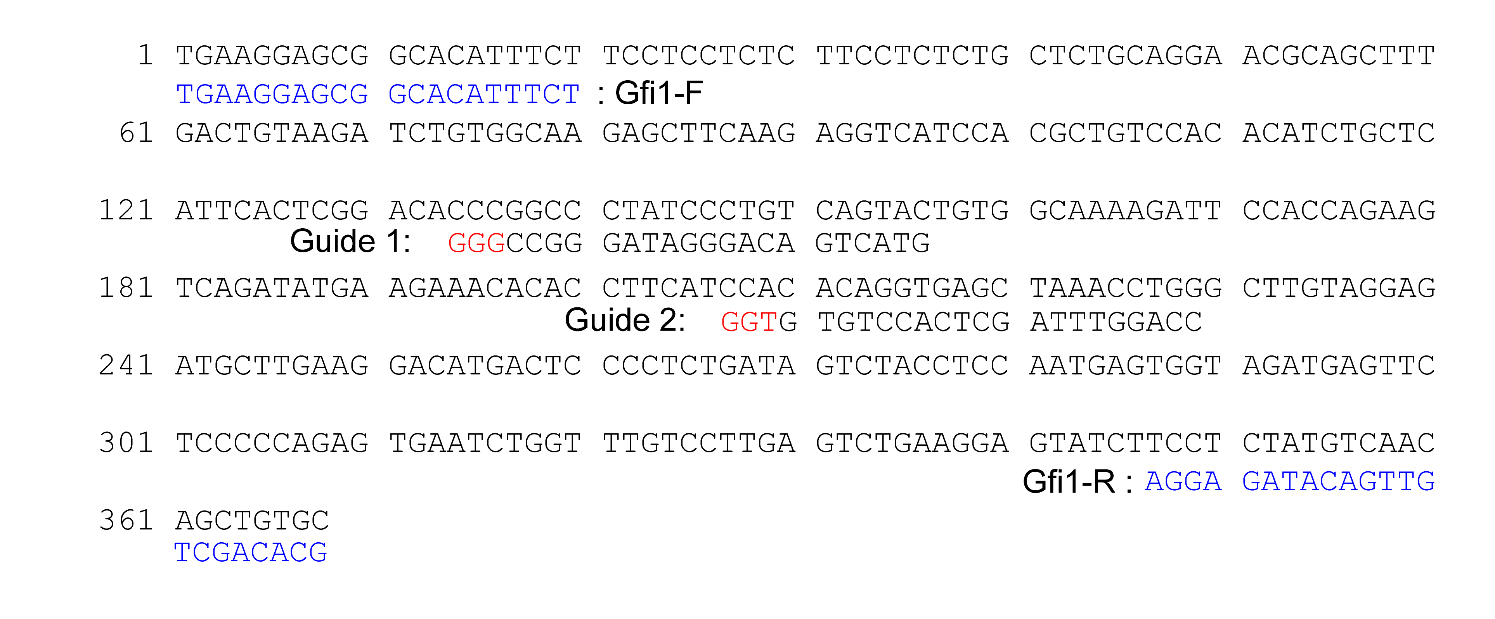


**Supplementary Figure 1** | The sequence information of CRISPR/Cas9 Guide 1 and Guide 2 targeting the mouse *Gfi1* locus.

The sequence of the 368-bp amplicon by performing PCR with primers Gfi1-F and Gfi1-R are shown and the locations of the two guides targeting *Gfi1* are indicated as Guide 1 and Guide 2. The protospacer adjacent motifs (PAMs) sequences are indicated in red letters, respectively.

## Supplementary Figure 2


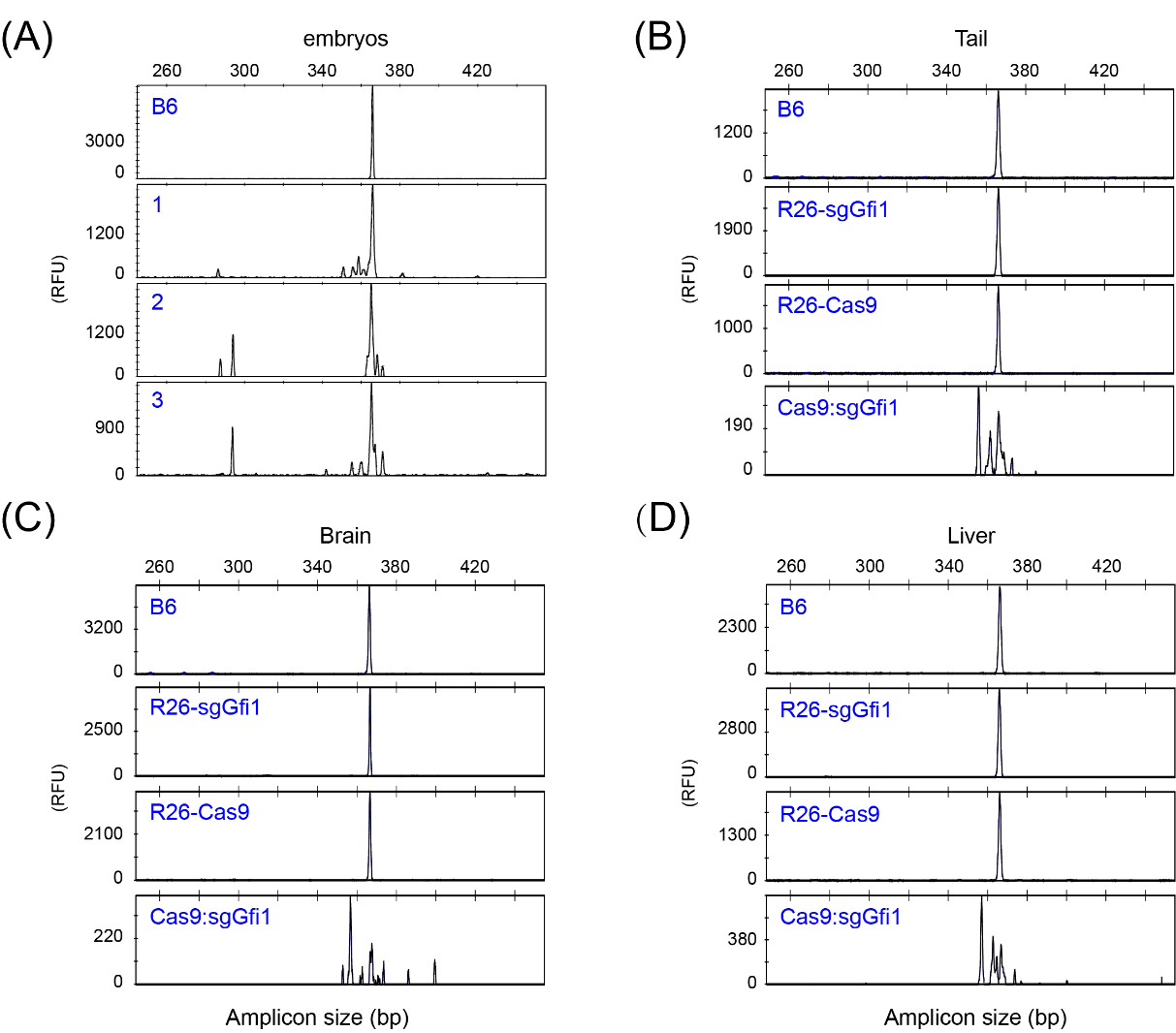


**Supplementary Figure 2** | Detection of CRISPR/Cas9-mediated genetic modification of *Gfi1* gene by CAE analysis.

**(A)** 18 zygotes were obtained from IVF using sperms from *Rosa26^U6-sgRNA-Gfi1^* mice and eggs from WT B6 mice and microinjected with Cas9 mRNA at 6-8 h after IVF. Single embryos were analyzed by fPCR-CAE 5d after IVF for detection of Genetic modification of *Gfi1* gene. Representative profiles (1, 2 and 3) showed shifted peaks compared to embryos derived from IVF using both WT B6 males and females (B6). (**B**−**D**) CAE profiles of genetic modification in *Gfi1* gene of Cas9:sgGfi1 mice, using genomic DNA samples of tail biopsies **(B)**, brain **(C)** and liver **(D)**. WT B6, *Rosa26^U6-sgRNA-Gfi1^* (R26-sgGfi1) and Rosa26-Cas9 mice (R26-Cas9) were used as negative controls. RFU, relative fluorescent unit.

## Supplementary Figure 3


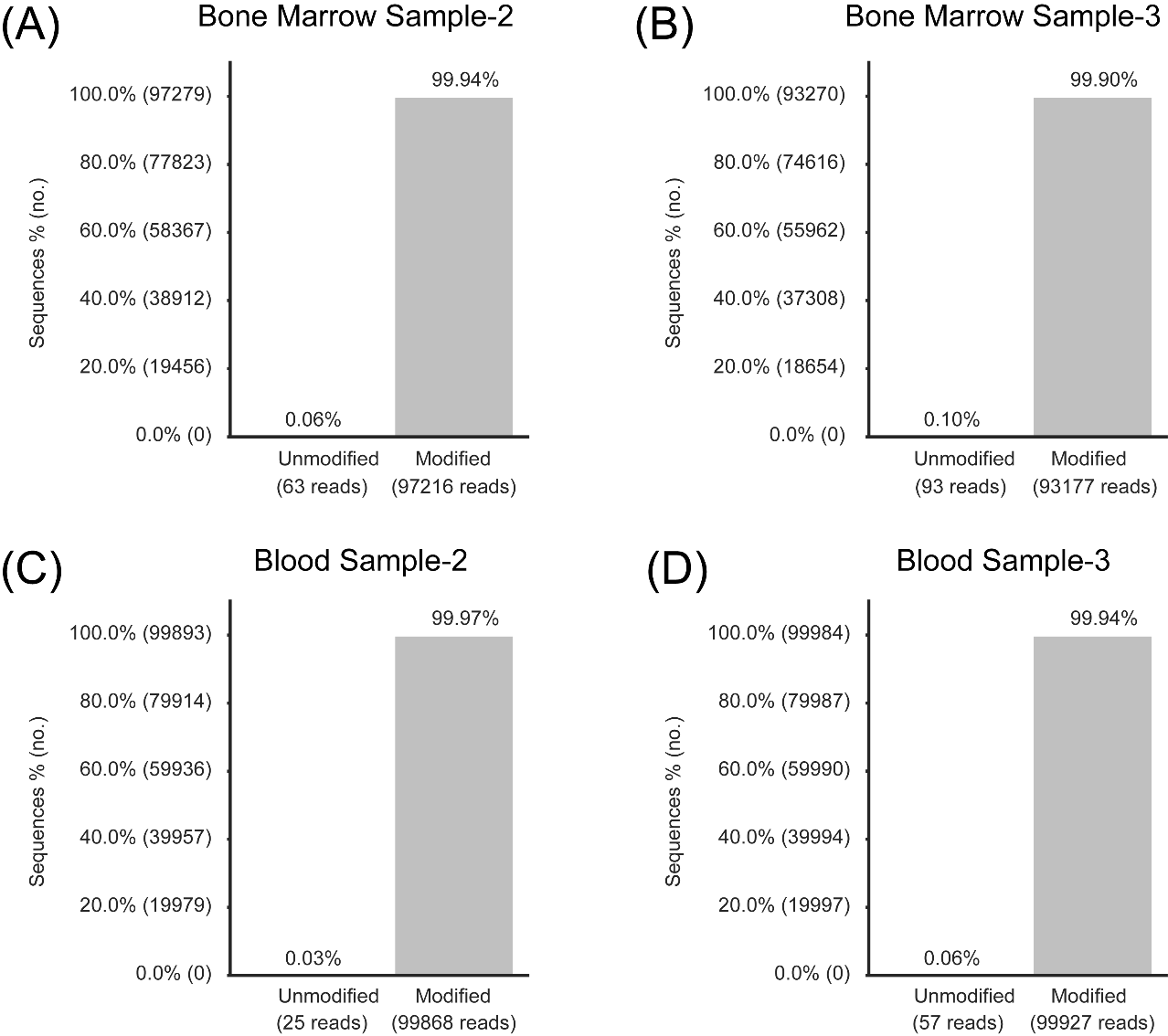


**Supplementary Figure 3** | Frequency of CRISPR/Cas9-mediated genetic modification of *Gfi1* gene identified by NGS analysis.

**(A,B)** The other two of the three bone marrow samples were used for analysis of DNA modification in Gfi1 targeted Cas9:sgGfi1 mice. **(C,D)** The other two of the three peripheral blood samples were used for analysis of DNA modification in Gfi1 targeted Cas9:sgGfi1 mice. The PCR primers Gfi1-F and Gfi1-R were used to amplify a region spanning Gfi1 targeted loci by Guide 1 and Guide 2, and the PCR products were analyzed by NGS to detect both unmodified and modified sequences by alignment of the reads to wildtype reference.

## Supplementary Figure 4


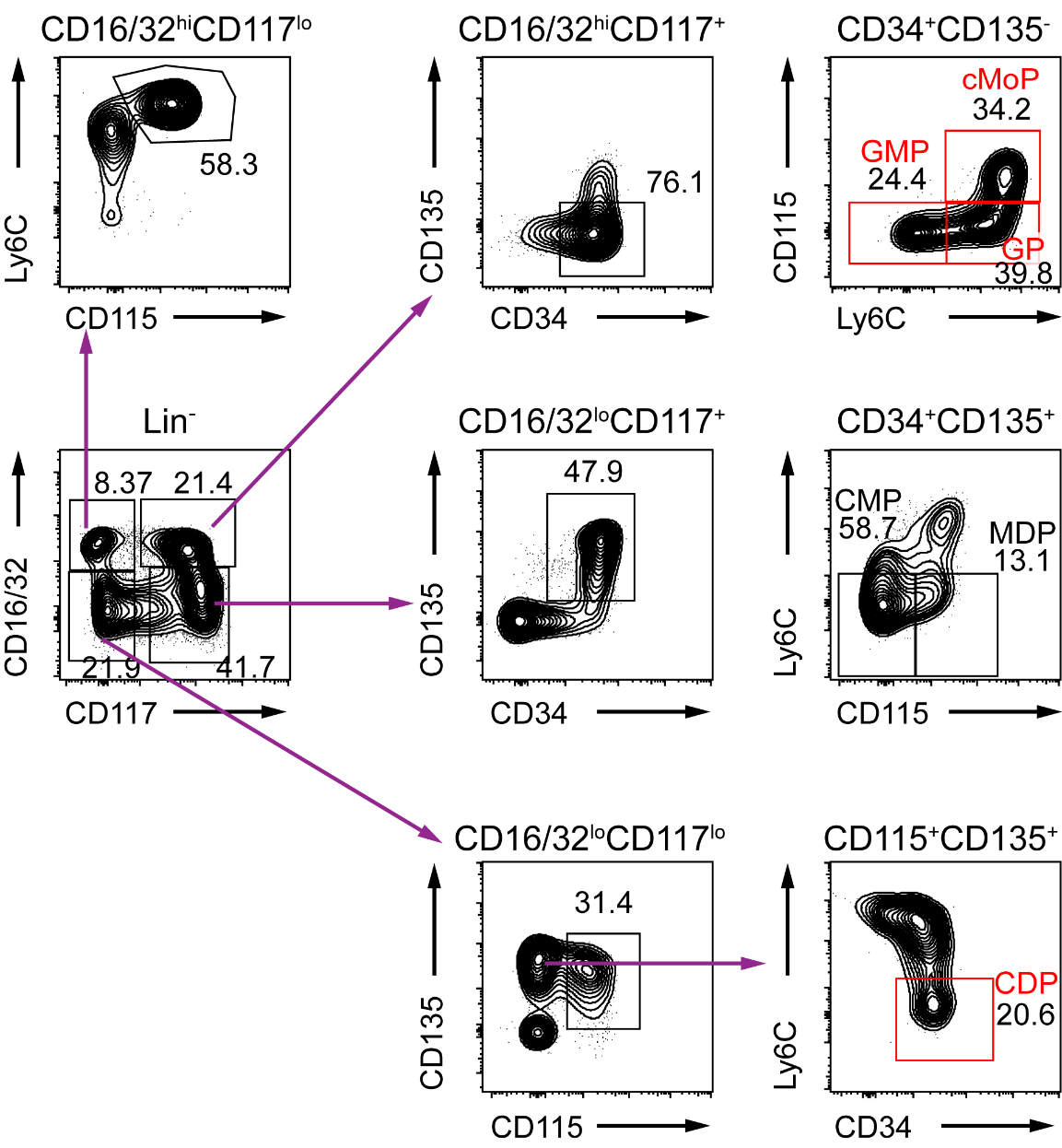


**Supplementary Figure 4** | Gating strategy of bone marrow (BM)-derived myeloid progenitors used for statistical analysis in **Figure 4B**.

Gating strategy used by Liu et. al (Liu et al., 2019). CMP, common myeloid progenitors; cMoP, common monocyte progenitor; MDP, monocyte-DC progenitor; CDP, common DC progenitor; GMP, granulocyte-monocyte progenitor; GP, granulocyte committed progenitor. Representative flow cytometric plots of CDP, GMP, cMoP and GP analyzed in **Figure 4B** were highlighted in red outlined areas.

Liu, Z., Gu, Y., Chakarov, S., Bleriot, C., Kwok, I., Chen, X., Shin, A., Huang, W., Dress, R.J., Dutertre, C.A., Schlitzer, A., Chen, J., Ng, L.G., Wang, H., Liu, Z., Su, B., and Ginhoux, F. (2019). Fate Mapping via Ms4a3-Expression History Traces Monocyte-Derived Cells. *Cell* 178, 1509-1525.e1519. doi: 10.1016/j.cell.2019.08.009

## Supplementary Table 1 | Primers used in this study.

| **Primer name** | **Primer sequence (5’ > 3’)** | **Usage** | **labeling** |
| --- | --- | --- | --- |
| Hprt-F | CGTCGTGATTAGCGATGATG | housekeeping gene used in real-time qPCR |  |
| Hprt-R | ACAGAGGGCCACAATGTGAT |  |  |
| Guide-1-F | TGACAGGGATAGGGCCGTT | detect the expression of Guide 1 by real-time qPCR |  |
| Guide-R | CCGACTCGGTGCCACTTTTT |  |  |
| Guide-2-F | GGTTTAGCTCACCTGTGGTTT | detect the expression of Guide 2 by real-time qPCR |  |
| Guide-R | CCGACTCGGTGCCACTTTTT |  |  |
| Gfi1-F | TGAAGGAGCGGCACATTTCT | produce a 368-bp amplicon for NGS |  |
| Gfi1-R | GCACAGCTGTTGACATAGAGGA |  |  |
| FAM-Gfi1-F | TGAAGGAGCGGCACATTTCT | detect indels of *Gfi1* by fPCR-CAE | 5'-FAM |
| RT-ssDNA | GTAAGCAGTAATCAATACCATG | synthesis of ssDNA by reverse transcription |  |
| 5’ external | AGAAGAGGCTGTGCTTTGGG | F0 genotyping PCR: span the 5’HA |  |
| 5’ internal | GGTGTTTCGTCCTTTCCACAAG |  |  |
| 3’ internal | AAGGACGAAACACCGCCAG | F0 genotyping PCR: span the 3’HA |  |
| 3’ external | GCCAGTCCAAGAGAAAGCACT |  |  |
| integration-F | GAGTTCTCTGCTGCCTCCTG | F0 genotyping PCR: span the integration of knock-in |  |
| integration-R | ACCTGTTCAATTCCCCTGCAG |  |  |

## Supplementary Table 2 | sgRNAs used in this study (NGG PAM site is indicated in bold).

| **sgRNA name** | **Sequence (5’ > 3’)** | **Usage** |
| --- | --- | --- |
| *Rosa26*-sg1 | CTCCAGTCTTTCTAGAAGAT **GGG** | targeting mouse *Rosa26* for CRISPR/Cas9-mediated knock-in |
| *Rosa26*-sg2 | CGCCCATCTTCTAGAAAGAC **TGG** |  |
| Guide 1 | GTACTGACAGGGATAGGGCC **GGG** | targeting mouse *Gif1* for gene knock-out |
| Guide 2 | CCAGGTTTAGCTCACCTGTG **TGG** |  |

## Supplementary Table 3 | Antibodies and fluorescent dyes used for flow cytometric analysis in this study.

| **Reagent** | **Clone** | **Supplier** | **Cat. No.** | **Source** |
| --- | --- | --- | --- | --- |
| CD45 APC-eFluor 780 | 30-F11 | eBioscience | 47-0451-82 | **Figure 2A,B Figure 4A** |
| CD5 PE-Cyanine7 | 53-7.3 | eBioscience | 25-0051-81 |  |
| CD11b Super Bright 600 | M1/70 | eBioscience | 63-0112-82 |  |
| CD3e FITC | 145-2C11 | eBioscience | 11-0031-86 |  |
| CD19 PE | 6D5 | Millipore | MABF311 |  |
| Ly6G Alexa Fluor 700 | 1A8 | BD Pharmingen | 561236 |  |
| CD45 APC-eFluor 780 | 30-F11 | eBioscience | 47-0451-82 | **Figure 4C** |
| Ly6G Alexa Fluor 700 | 1A8 | BD Pharmingen | 561236 |  |
| CD19 PE | 6D5 | Millipore | MABF311 |  |
| CD11b Super Bright 600 | M1/70 | eBioscience | 63-0112-82 |  |
| CD16/32 APC-CY7 | 93 | Biolegend | 101328 | **Supplementary Figure 4** |
| CD34 BV421 | SA376A4 | Biolegend | 152207 |  |
| Sytox Blue Dead Cell Stain |  | ThermoFisher Scientific | S34857 |  |
| CD115 Super Bright 600 | AFS98 | eBioscience | 63-1152-82 |  |
| CD11b PerCP-Cyanine5.5 | M1/70 | eBioscience | 45-0112-82 |  |
| CD11c PerCP-Cyanine5.5 | N418 | eBioscience | 45-0114-82 |  |
| LY6G PerCP-Cy5.5 | 1A8 | Biolegend | 127616 |  |
| CD49b PerCP-Cy5.5 | HMα2 | Biolegend | 103519 |  |
| B220 PerCP-Cyanine5.5 | RA3-6B2 | eBioscience | 45-0452-80 |  |
| CD3e PE-Cyanine5.5 | 145-2C11 | eBioscience | 35-0031-82 |  |
| CD19 PerCP-Cyanine5.5 | eBio1D3 | eBioscience | 45-0193-82 |  |
| TER-119 PE-Cyanine5.5 | TER-119 | eBioscience | 35-5921-82 |  |
| CD135 Biotin | A2F10 | eBioscience | 13-1351-82 |  |
| CD45 Alexa Fluor 700 | 30-F11 | eBioscience | 56-0451-82 |  |
| CD117 APC | 2B8 | eBioscience | 17-1171-82 |  |
| LY6C PE | HK1.4 | eBioscience | 12-5932-82 |  |
| Streptavidin PE-Cyanine7 |  | eBioscience | 25-4317-82 |  |
